# The meaning of fatherhood to men in relationships with female sex workers in Kampala, Uganda: The struggle to model the traditional parameters of fatherhood and masculinity

**DOI:** 10.1371/journal.pone.0273298

**Published:** 2022-08-31

**Authors:** Martin Mbonye, Godfrey Siu, Janet Seeley

**Affiliations:** 1 MRC/UVRI and LSHTM Uganda Research Unit, Entebbe, Uganda; 2 Global Health and Development Department, London School of Hygiene and Tropical Medicine, London, United Kingdom; Muhimbili University of Health and Allied Sciences, UNITED REPUBLIC OF TANZANIA

## Abstract

Many women who engage in sex work in sub-Saharan Africa become pregnant, often unintentionally. There is limited attention paid to the experiences of fathers of children born to women engaged in sex work. We examine the meaning of fatherhood to these men, the significance of children, and how they navigate the economic and cultural challenges of fatherhood in this context. Analysis is based on ethnographic data from 13 men who identified as intimate long-term partners of female sex workers (FSW) in Kampala City, Uganda. Our findings illustrate how men who have children with FSW struggled to model the traditional parameters of fatherhood and masculinity. We found that men who had children with FSW faced hurdles fitting within the social construction of ideal fatherhood. Accepting fatherhood often started with doubts over the pregnancy because of the multiple partnerships of women. Men who only saw themselves as clients struggled to adjust to being fathers because of their perceptions of the social implications of fathering a child with a FSW. Integration of mothers who were also sex workers into the man’s extended family was a challenge because of the fear of negative reactions from family members. However, when men accepted their roles as fathers, they started seeing value in their children. Due to poverty, most of the men fell short of the societal measures of masculinity, but children transformed their social status before their society and family. The provider role often used to define good fathering was a challenge for men. However, the financial support from FSW partners softened the burden and facilitated the creation of a family environment constructed to the perceived standards of the broader society. Our findings provide insights into the state of parenting among FSW and their partners which can guide interventions that are tailored to their unique circumstances.

## Introduction

The number of women in sub-Saharan Africa who become pregnant, often unintentionally, while engaging in sex work is high [1–3]. There are, therefore, many clients of female sex workers (FSW) and other men who father these children, at times without being aware of the fact and the responsibility [2]. Some attention has been paid to the experiences of these men as intimate partners or clients [2, 4], but little on their role as fathers.

In different African cultural settings the fatherhood role is defined by gendered expectations of roles [5–7]. In central Uganda, fatherhood is associated with respect and responsibility; a prestigious identity to have [8, 9]. Historically children among the Baganda, the dominant ethnic group in central Uganda, belong to the family lineage of the father [10]. The father’s brothers, the paternal uncles, act as ‘other’ fathers to the children, imbuing the father’s role with expectations of protection, of the child but also of the clan the child has been born into [10, 11]. A child that is born in a union that is not recognized culturally or religiously, because no ceremony has linked the parents, is brought into a world outside this system, unless the father claims the child, and his clan identity passes to his child.

The context in which one becomes a father matters in shaping how a father approaches the role [12]. For those who have a child with a FSW, the identity of the mother certainly plays a part in shaping how such fathers approach their role, and how others in the society perceive them. If the FSW mother establishes an intimate relationship with the father of her children and they organize their family in accordance with local social systems, then the children have the security of the structure of that system [12, 13]. However, when a child is born as a result of paid sex, with a woman who is a sex worker, the child may be shunned by the father, particularly if he does not believe that he is responsible for the pregnancy, and because of the stigma associated with sex work [14, 15]. The future for that child may be insecure, particularly if their single mother struggles to make a living and continues to face the dangers associated with sex work [16]. Sex work vulnerability may even increase as FSW, who tend to be abandoned face the need for more income and therefore may intensify their involvement in sex work and take more risk for extra money [4].

Men who acknowledge that they have fathered a child with a sex worker do, nevertheless, find ways to fit the children and the mothers of those children into their lives [17]. Previous research has shown that while men who establish relationships with sex workers find meaning in these sexual partnerships, they can also face a dilemma over the nature of that bond in viewing a woman both as a provider of paid sex, but also a long-term partner [18, 19]. This may affect how such men engage with their social roles as fathers who share the parenting role with women in sex work. The provision of sex as part of a transaction with a client is generally accepted as being non-reproductive [20]; where a pregnancy does occur the woman may face, and accept, the denial of paternity and abandonment by the father of their child [21, 22] or, if a man does accept the child, there are limitations on how that man engages with the social role as a father in sharing parenting with their FSW partner.

Research on parenting and sex work is limited, and tends to focus mainly on the experiences of women as single mothers [16, 23, 24], with little attention given to the experience of men who father children with female sex workers. In this paper we describe the experiences of men who become fathers to children of FSW. We examine the meaning of fatherhood to men who father children with FSW, the significance of children, and how these men navigate the economic and cultural challenges of providing for their families, juxtaposed with the social stigma associated with sex work. This study addresses an important gap in the literature on the state of fatherhood among men who have children with women in sex work. We aim to shed more light on the involvement of these fathers in the lives of children that are born to female sex workers.

## Methods

### Study setting

Kampala city located in central Uganda has a population of approximately 1.5 million people [25]. Kampala is divided into five political divisions and the study was conducted in two of those divisions. Based on observations as part of the ethnographic aspect of the study, the lead author noted that the two divisions share similar characteristics to other divisions with a few variations. They are densely populated, most of the residents earn low-wages and typically reside in single rooms that are not well constructed, often with poor drainage, as these offer cheap accommodation [8].

Economic activities tend to be concentrated alongside the main roads where shops, commercial taxi stages, bars and restaurants are located. In such places, the work available to the urban poor gives them few chances of a sustainable income. Many of the men and women are engaged in bar work, hawking products for large businesses, taxi and commercial motorcycle (locally called boda-boda) transport, construction labour and similar unskilled and low paying seasonal employment. During the night-time, the biggest businesses that are visible are those associated with entertainment in the form of night clubs, bars, restaurants, and lodges. There is almost unlimited access to alcohol with some selling points open day and night. Some of the unregulated alcohol sales venues hide these services by selling other items. During observations it was noticeable that some of the retail shops had a section at the back where those interested in alcohol would gain access and engage in alcohol consumption.

Selling sex is a common activity. A few brothels that are hidden from public view exist where sex workers rent rooms and conduct business at any time, but mostly at night. Sex workers prefer locations in and around night clubs, guest houses/lodges and dark streets. Sometimes the police interfere with sex work, conducting raids and arresting sex workers, and on rare occasions their clients, since sex work is illegal in Uganda [26]. It is also illegal to sell and to provide venues where sexual services are offered which means it is done often under cover and often in disguised forms [3, 26].

### Study participants, sampling and data collection

We conducted repeat in-depth interviews with thirteen men who were involved in relationships with FSW. These data were augmented by observations in the different settings of the 13 men and with information from two focus group discussions with FSW who had children with non-commercial partners. The study was conducted from January to December 2019 in Kampala City. These13 men were partners of FSW who were attending a research clinic. This research clinic also known as the Good Health for Women Project (GHWP) clinic run by the Medical Research Council/London School of Hygiene and Tropical Medicine and Uganda Virus Research Institute is located in Kampala (MRC/UVRI and LSHTM Uganda Research Unit). The project started enrolling FSW in 2008 and by 2011, willing long-term male partners had been included [27, 28].

Female sex workers attending the GHWP were informed about this study and those with intimate male partners were asked to inform them about this study. Fifty women had agreed to contact their partners and from these 13 men were purposively selected. Seven of the thirteen men were registered members of the GHWP while the other 6 were not. In order to generate a sample with maximum variation among those men attending the GHWP, we used HIV status, residential and work patterns, time since they joined the GHWP and length of time they had been in a relationship with a sex worker, to purposively sample the men. For the men not attending GHWP, we used an opportunistic sampling approach by capitalising on opportunities where such men became available. We had notified FSW with such partners that we would recruit them whenever they were available for the study because some men had expressed interest but wanted to think about it or make up their minds at a later point. Therefore, such men would notify their FSW partners who would inform the lead author and he would initiate contact. Arrangements to meet each of those men who were not part of the GHWP were made at a place of their convenience but typically somewhere within their settings at which information about the study was shared with them.

Observations were conducted in the settings were the men resided, spent their leisure time and work places and conversations were held with the men and their significant others. During field observation visits, notes were made using a pocket size note book with care being taken not to interfere with the natural flow of activities. After the visit detailed accounts were typed onto a password protected laptop providing a detailed description of the field observations.

The observations were very important in informing the probing process during the in-depth interviews. After sufficient rapport had been established, the lead author conducted in-depth interviews with each of the 13 men and re-interviewed nine of the men at a later time. The other four men had travelled out of the study area by the time of the second interview. Visits were conducted at different times of the day or in the evenings and over the weekends to maximise the variety of experiences.

The first interviews were not audio-recorded. Audio-recording was not accepted practice at the GHWP, given participants concerns about having their voice on tape which could compromise their privacy [29]. The lead author took field notes during the interviews and conversations, which were later written up in detail. On average the duration of interviews was between 45 minutes and one hour. Adequate care was taken to conduct interviews in private places chosen by the participants. These were usually at their homes or in private spaces in restaurants during less busy times to enhance privacy. Later those with whom a second interview was conducted, verbally consented to having them audio recorded. The topics discussed during in-depth interviews focused on how men became fathers, the perception of men towards the fatherhood role, the meaning of fathering a child with a sex worker and integration of the child into the family of the man. Prior to the in-depth interviews, the lead author spent the first few months establishing rapport and getting to know the study setting. This helped put context to different aspects of life of the men that might have otherwise been missed if he had not taken this time to learn. This process helped in generating the topic guide that was detailed and that accommodated language that was well understood by the men from interacting with them. The semi-structured interview guide was therefore a product of the literature that informed the research questions and the lead author’s own interaction with the participants during field work.

We were keen to add the voices of FSW who had children with non-commercial male partners. In order to capture the FSW perspectives, two focus group discussions were conducted. One of the groups was composed of women that were registered with the GHWP while the other was with those who were not registered. To determine the size of the group, the lead author relied on his experience and based on the literature on conducting focus groups where it is suggested that a small size is sufficient for a group when discussing a sensitive topic [30]. We adopted this approach since the women were more comfortable talking in small groups. The first group discussion, made up of women attending GHWP, consisted of five members.

For the second group discussion, women who were not attending the GHWP were approached in order to compare their experiences with those that were attending in relation to the research aims. These women were recruited from a brothel where two of the male participants worked and spent time. These men helped in the mobilization process since they knew the women we were interested in talking to. The discussions were held in a private place that provided adequate freedom of expression. This group was made up of eight members.

In terms of the number of groups we drew on our own experiences and that of a previous study which examined the characteristics of different groups and concluded that a sample of two or three focus groups was found to capture 80% of the themes on a topic [31]. The topics discussed in focus groups centered on the experience of sharing parenting roles with intimate male partners, how gender roles were distributed within the household, the meaning of children in the relationship, how men perceived sex workers who were mothers and how they managed the social stigma associated with sex work within the family setting. The focus groups were conducted in the local Luganda language which the participants were all familiar with and audio recorded.

### Data analysis

Notes about emerging themes were taken by the first author, discussed among the co-authors and ideas for further enquiry shared. The particular theme of fatherhood emerged from participants’ accounts of their family lives and experiences as adults with children. The final analysis followed a systematic thematic approach as suggested by Braun and Clarke in their seminal paper [32]. We developed coding frames based on topics and summarised the data using excel tables to represent themes and subthemes. We generated themes from in-depth interviews and FGDs separately and later merged them, for this paper based on similarities and relevance to men’s experiences as fathers.

### Ethical considerations

Ethical approval for this study was obtained from the School of Medicine, Research and Ethics Committee, Makerere University College of Health Sciences (#REC REF 2017–155) and from the Uganda National Council for Science and Technology (#SS 4849). Written informed consent was obtained from the participants who participated in in-depth interviews and focus group discussions.

## Findings

### Participants’ characteristics

The 13 men were aged between 26 to 51 years and most had not completed primary level education, while a few had some secondary education and one had completed university. Almost all worked in what could be called the informal sector, with only four reporting a monthly salary. However, all were dissatisfied with their income and many spent large periods without earning or had to depend on others, including their FSW partners for support. Eight of the men described themselves as being married, often using the Luganda word ‘*Mukyala wange*’ which loosely translates as ‘my woman or my wife’ to describe their partners, while the rest reported having separated from their partner, who was known to be a sex worker, and reported being in a new relationship. There were a few of those who reported on and off relationships, with the shared child the main reason for keeping the contact. Except for two men, all had had a child with a sex worker in addition to children begotten with other women. Nine of the 13 men were ethnic Baganda but the non-Baganda men understood and spoke fluent Luganda which was the language used to collect the data.

There was a total of 13 female participants in the two focus groups, aged 24 to 49 years. Each woman was selected on the basis of being in a relationship in which they shared a child with a non-commercial male partner. The women were all engaged in brothel, bar and/or street based sex work or a combination of these. Ten of the thirteen women were from the Baganda ethnic group. All the women understood and spoke fluent Luganda. Three of the women did other jobs such as saloon work and food vending in addition to sex work. All the other women were full time sex workers. None of the women was married to their non-commercial partners but most reported cohabiting or receiving support from that partner.

In presenting the findings we use three summarized cases to illustrate important themes and sub-themes that reflect experiences of fathering a child with a sex worker. The cases are used to illuminate each theme but we also draw from the experiences of other men as we discuss the different themes. The three major themes that we discuss take us through different paths in which men accept their roles as fathers, starting with reactions to the pregnancy of the FSW. We then explore the meaning of children to the men and how they impact on their private and social lives. Lastly, we discuss the theme of providing for the children and the non-providing aspects of fatherhood. We use pseudonyms for the men to protect their privacy and confidentiality.

### Accepting the fatherhood role

Accepting the role of a `father’ was a challenge for many men because of the circumstances under which they had become fathers. For many, it was a struggle against the ideal way to become a father. We describe Donald’s experience to illustrate how this issue was experienced:

Donald, was a 45 years old university graduate. Despite this level of education, he did not have a job that offered a stable income and spent a lot of the little he earned on alcohol and women. Donald enjoyed the company of his peers and in the evenings was usually at one of the nearby bars drinking alcohol and targeting women for sex. This is how he met his current partner when he became a regular client of hers and started condom-less sex. Donald called this relationship a casual pleasure-seeking affair and never had any plans to progress beyond that level. Such was his shock when she announced that she was pregnant. So many thoughts went through his mind including denying responsibility and shunning her, but his conscience told him to take on the responsibility. This included looking for money to help with the delivery of the child, providing for some of her home needs and at times contributing to her rent. Donald was aware of the social scrutiny he faced and had already received comments from a friend who had told him that he was risking infection with HIV if he did not stop interacting with the FSW. He regretted the ‘mistake’ he had made, and it took him a while to reconcile with his position as a long-term partner rather than a client. It was not until his father commended him for bringing a boy child into the family that Donald started treating his partner in a more respectful way. He even started calling her mother of my children as opposed to the previously degrading reference to her as ‘*one of those women*’. With time Donald and his partner settled into a long-term relationship and had two more children, including one born during the field work period.

To men like Donald, fathering a child with a sex worker was unexpected, unwanted and shameful; and was less than an ideal way to become a father because the only link he had intended with such women was for sexual purposes only. This is a view that was echoed by women in the group discussions. Women in the focus groups reported how easy it was for men to shun their responsibilities when they found out about a pregnancy. One woman commented:

*I once had a long-term partner but when he finished making me pregnant he found another woman. He then later returned after I had aborted when he left me. So when I had aborted he returned to me after his problem had been sorted and wanted us to resume the relationship but I refused*.

When Donald attempted to deny responsibility of the pregnancy, he was following what most men were accused of doing, abandoning the sex workers to raise children on their own. In some instances, Donald described how he did not want to be like those men, but he also never wanted the label of sex work to be associated with his family. His initial denial of responsibility can therefore, be seen through the stigma lens. In earlier conversations with the first author he had described how he had envisioned marriage with a decent, educated woman from a respected family like his own. His fatherhood story was a contradiction of the one he had anticipated. This experience as narrated by Donald highlights the double standards of men who enjoy sexual pleasure provided by FSW yet struggle with a pregnancy that may result from the sexual encounters.

Relatedly, men’s perception that FSW were likely to have multiple partners created doubt over paternity. For some, there was real evidence to justify their doubts. Such was the experience of John 40, who had been in a long term relationship with his partner but was also aware that she had another male partner. His partner had admitted that she was not sure that he was the father of ‘their’ daughter. It took a lot of convincing for John to eventually accept he was the father of the child. His change of mind was assisted by the resemblance the child had with him that he had started noticing as the child grew up and counseling he had received from a church leader:

*It was after the intervention of the pastor that I got convinced that the child is mine. The pastor asked me to look at the child’s forehead and indeed the forehead looks just like mine*.

### Integrating the sex worker into the extended family

Once a sex worker became a mother, men who could not severe the relationship, with time, thought about the best way to integrate the sex workers into their extended families. Men were aware that this would not be an easy task given the strong social stigma against sex workers and the moral questions involved. Donald reported having been discouraged by some of his relatives when they learned that he was in a relationship with a sex worker. The arrival and/or disclosure of a child facilitated a woman being accepted in the husband’s family, but men also had to find additional reasons to rationalize the relationship. Strategies included keeping silent about the work the woman did or presenting a different identity such as working in a bar, while others emphasized the mother role to divert attention away from sex work. For some women, their access to money and other opportunities ensured they were in a strong position to receive approval of the partners’ relatives, while others were provided the much-needed support and care to the men who had become problematic or a care ‘burden’ to the families, such as those who were alcoholic or sick.

Some men, however, worried about their health and how their partners would react once they found out that they could be infected with HIV. For example, many men had suspected HIV infection or had known about being HIV positive but had not disclosed to their partners fearing abandonment. It was especially true in those relationships in which the male partner depended on the FSW for support. For instance, the partner of Sula (aged 39 years) found out about his HIV status when he was admitted to hospital following a serious sickness. It was his mother who inadvertently disclosed to his partner when she thanked the partner for sticking with Sula despite his HIV positive status. Sula’s partner was surprised that his HIV status was known within his family and yet she knew nothing about it. Although this disturbed her she had made up her mind to stay with him for the sake of the child that they shared even though she was HIV negative:

*At the hospital when he was admitted his mother came to help care for him and that is when she told me about his HIV story and appreciated me for sticking by him despite his status*.

In the case of other men, integration was smoother because of the prevailing social and economic circumstances of their partners. For example, John introduced his partner to his parents who were staying a long distance from the city and they received her with joy. They thanked her for helping convince him to start treatment for the HIV related ailments that were beginning to worry them. Additionally, Stuart 37, had for a time depended on the hard work and support of his partner and had come to be proud of her for these qualities. She compared a lot better than the other women he had left behind in his village and was committed to a long-term relationship with her in Kampala where he had few relatives.

Nonetheless, full integration into the extended families remained a common challenge for the sex workers. Their identity, alcohol consumption, and for others, assertiveness and aggressiveness, appeared to be major obstacles as these attributes brought disrespect/ disrepute to the families. Fred 51, always felt ashamed of his partner when he heard that she had been involved in a fight or had drunk herself to a stupor which restricted his desire to fully associate with her in family circles. Clearly, Fred found these behaviours unfit for a mother and wife. His view was shared by some women (FSW). For example, in focus groups, although some women felt at ease with their partners knowing that they were in sex work, many felt that it was so shameful to expect any man to proudly associate with a sex worker and so they understood that integration into the man’s family would be selective. This is summed up by one of the women from one of the FGDs who thought that the work they did could not permit full acceptance and therefore integration into the family:

*…But for us if a man finds you in this work [sex work] even if he says that he loves you he is not being truthful. He would rather love some ugly women than be seen walking around with you as he feels ashamed because you are a common property [entertain multiple sexual partners] and he cannot control you*

### Meaning of children

After navigating the hurdles and embracing the fatherhood role, men turned attention to what the children meant to them. Their accounts show how having children in the context of a relationship with FSW conveyed different social meanings to the men. While for a few men in this study, getting a child transitioned them to adulthood and was an important resource for constructing a desired masculinity, for others, children and fatherhood in this circumstance was a highly contested matter both by themselves and by the partner. Patrick whose story we recount below was a good example.

Patrick was a 37 year old taxi driver, who was highly respected among peers for having been one of the few senior drivers who owned his own taxi. He was reputed for his physique, having been a boxer earlier in his life. Patrick liked spending time in bars, from where he subsequently met a woman who happened to be selling sex. Their 3-year-old relationship resulted in two children, for whom he was proud as they had given him a respectable identity as a father which in his clan and among fellow men was a prestigious thing and a sign of maturity. However, following a period of tension that led to Patrick separating from his partner, he assumed that he would get possession of the children in line with cultural expectations that children belonged to the man and his lineage. But his partner surprised him when she resisted his attempt to take custody of the children. This was unexpected challenge to his masculinity, and Patrick claimed he ‘did not know how to react’. In order not to further ‘injure’ his reputation, Patrick avoided physical violence against his partner, in spite of being a renowned former boxer. Although Patrick resigned and let her ‘take’ the children, he decried the implication of this to his masculinity, and described himself as having been left feeling powerlessness, and not man enough. He decided to relocate from the area to avoid shame in case others got to know about the story and ‘never’ to discuss it with peers for fear of losing his masculine reputation.

Patrick’s story, like that of several other men, shows that having children was associated with a certain social status and bestowed a positive identity. However, the contestations and doubt about paternity and/ or custody of children that characterized these relationships, resulted in him being dispossessed of children and significantly threatened the sense of masculinity attained through fatherhood. However, while, many men would have opted to simply walk away from such a situation of contestation over children with FSW, Patrick tried to fight for custody only to face unexpected resistance. Patrick’s experience also illuminates the agency women had in matters concerning their children. This was also reflected in the case of another male partner of a FSW who lost his fatherhood rights in the most unexpected circumstances. Musa was a 49 year old man whose relationship with a FSW had lasted nine years. In that time together with his partner, they had had twins, who brought a lot of joy to him that he publicly acclaimed his achievement as a father of twins. Musa, like other fathers of twins, earned a respectable status in society and enjoyed the title of a *salongo*, given to a father of twins. However, after nine years events took a drastic turn when Musa and his partner had disagreements and she decided to leave him. Before leaving, she disclosed to him that he was not the biological father of the twins and therefore had no rights over the children. While this revelation brought back a painful childhood memory and fear that he would never have children due to an injury he sustained when a ball struck his genitals, he decided not to contest the allegation as he was uncertain of his virility. However, because the twins had become an integral part of his identity, Musa struggled to recover from the emotional and psychological blow it dealt him:

*I recall a painful moment in my life when my previous partner lied to me that the two children I thought were mine actually belonged to another man. I felt betrayed and asked myself that how can I look after someone else’s children for nine years thinking that they are mine only to learn that they are not?*

In general however, once they embraced their new status as fathers, men also reflected on the health benefits that accrued. For some, fatherhood prompted changes in behavior. Raymond, a 35 year old man commented: *‘I took the [HIV] test because I looked at my children and felt that I should be in their lives and help them grow’*. When he was found to be living with HIV he started treatment in order to remain healthy and participate in the lives of his young children.

A further benefit described by men was that, children led to rethinking about the status of the relationship with the sex worker. Clients like Donald were forced to redefine their perceptions of the sex worker who had become a mother of their children rather than a woman who offered sexual services. Long term partners of sex workers, like Jimmy (aged 38), mentioned how the pregnancy had prompted discussion of marriage, although there was a condescending tone to his statement: `*she keeps demanding for a wedding like all women’*. This tended to happen at a time when a woman was pregnant and wanted stability with the male partner. In other cases, having a child with a sex worker seemed to endear the men to their partners, and created a deeper bond that helped the relationship survive challenges. William, the youngest of the men at 26, had for a while depended on his partner for accommodation but he had been thrown out of the house after a disagreement and was homeless. However, this did not stop her from occasionally helping him because of the child they shared:

*…but when I have a problem she comes in and helps when am in a desperate situation and when I fall sick she allows me to stay at her house but when I am not sick she cannot allow*.

Nonetheless, there were accounts from the participants describing how children begotten in these partnerships had complicated and/ or severed their relationship. Women in focus groups described how common it was to have absentee and or uninvolved fathers, as men responsible for pregnancies tended to deny responsibility or were not easy to identify. Mothers also described frustration with absent fathers, especially when children questioned them about their paternity:

*…My child even asks me whether it is true that the father is really the father. The child has reached Senior three and everyone says that the chairman’s child resembles him, but the child asks why does he not care about us?*

### The predominance of the provider role

Being a provider was perceived by the men as the most important of all the roles of a father, and this narrative, often overshadowed the discussion about other parameters of fatherhood. In Raymond’s story we show the attention men and women gave to this role and how they downplayed other roles, such as parental bonding, love and moral guidance to the children.

Raymond was a 35-year-old man who had two children with his partner. He had met his partner when they were still at school but had gone separate ways when both he and his girlfriend dropped out. Raymond describes dropping out of school as more of his choice rather than circumstantial as he still had support to continue with education. He had lost both parents to what he describes as AIDS related sickness. The mother died first and the father followed shortly after. After his parents’ death he said. *‘I joined bad groups and never cared about AIDS*. He got his first child from a relationship where he also believed he contracted HIV. Later he met again with an old girlfriend who was now in sex work and they started a relationship. She disclosed to him that she was HIV positive when she had their first child and encouraged him to test but he hesitated. Deep down within him he suspected HIV infection but was scared of confirming it. However, when he had the second child with this partner, he started worrying that he might die and miss participating in his children’s lives. He looked at his children, and his partner and noticed how her health was improving, because she was on treatment, and decided to test. He shifted attention to the family when he started treatment. His conversations were now focused on a future with his children. He loved to talk about milestones in his children’s lives and even rented a house in a relatively expensive residential area arguing that; *‘the children should grow up with rich neighbors who will help them in future*’. It was like an investment. He applied a lot of the parenting style of his father which involved consulting children over some matters and ensuring personal involvement in education and socialization. He intended to keep the balance even though he admitted that whenever he could not provide, he felt uncomfortable as a man. He has started noticing improvements in his health and is happy that the feared stigma never materialized. He is able to continue his routine with relatively few noticeable interruptions in his life which gives him comfort.

Both the women and the men often viewed a father as being a provider, drawn from the predominant social construction of fatherhood in the community. Providing fathers felt that they had done enough to qualify to be regarded as good fathers while those who did not provide, found themselves facing social ridicule. The importance placed on this role by both men and women as well as the wider society, tended to over shadow the other attributes of fatherhood. Men like Raymond [35] and John (aged 40), described and valued other roles such as being able to spend time to play with children and talking about health and giving advice that would mold children into admirable characters with good values. Yet even for men like these, the provider role remained the gold standard as a measure of a successful father. Similarly, John (aged 40) who sometimes enjoyed playing with his daughter and often spoke about her health and happiness still felt that without providing, there was little he could point to as being a successful father. He downplayed his non provider roles by saying that: *‘like any other man*, *I have to look for something to feed my family’*. He, like many other fathers, described the sense of relief they usually felt on the rare occasion when they would get some money and managed to afford the more expensive food, particularly meat. These men were uncomfortable that they could not provide consistently and had to depend on others, particularly their partners to fulfill this highly cherished role. In John’s words, the pain of what he calls failure to perform his role as a man is illustrated in the following quote:

*As a man I know that my responsibility is to take care of my family and sometimes I try my best to do this but the money sometimes becomes difficult to get. Well, this woman (FSW partner) has been such a blessing to me. She has been very patient with me and always supports me. Like now when I got this attack and broke my finger and almost lost my life, she has been the sole provider at home and was able to give me money for my hair, to treat this wound and take care of the home. I am not happy that she has to play my role. It makes me very sad*.

Yet, this quote, like many others from our participants, not only describes men’s sense of failure, but also it makes it clear that the female sex workers bring significant benefits to the male partner’s lives. The FSW partners bring sexual pleasure and satisfaction, which was how the relationships started, money and financial support which in some cases resulted in a higher standard of living, lodging assistance when needed, such as in the case of William (aged 26), as well as support and care for illness, including HIV and related health issues, and the higher social status and positive identity conveyed upon men by being a father. However, that said, these benefits are tainted due to the stigma of sex work.

Some women appreciated that their partners might not always be in a position to provide. They were happy to assist the fathers of their children if they felt that the men were doing something to find work and assume their position as the provider. In the focus group discussions one woman whose views were supported by the other group members observed:

*Now when I take over his responsibilities of buying food, paying rent, taking care of the children, feeding them and dressing them. So if I have accepted that my husband is poor, I can go and we work and with time things might start improving and if God blesses you as the man you may pick up and get some good income and resume your duties as a man*

The men who provided seemed to be more confident about performing other roles as well compared to those who rarely provided. They talked of the care and attention they paid to the children they had besides providing. Fred, a 51-year-old man, was proud to be able to provide for a family of more than 20 children including non-biological children he had `adopted’, and three women, but he also saw moral training as his role, particularly given the widespread negative peer influence and immorality in the urban environment:

*…we built a wall for them with a gate…So for me I talk to them [his children] since we grew up also being advised. So, I make sure that they don’t even go to the rooms of those people who are renting nearby, they only rotate around here even though the other side, there are also children…Yes, you as a parent I have a role to play to make the child learn good manners*.

Mothers discussed moral training and father role modelling of children in light of sex work. They particularly blamed fathers’ failure in this role for the increased number of children vulnerable to sex work. They described being abandoned by the fathers of the children and the impact was that those children did not have any fathers to impart proper morals:

*Once the men became lazy, and they stopped looking for money and taking care of the home that is when we also started to go out to look for money. So we are now overwhelmed with working and we fail to impart values in the children and so the children have now become spoilt*.

## Discussion

In this paper, we explored the experiences of fathers of children whose mothers are sex workers in Kampala. We examined, experiences, processes and struggles men go through as they come to terms with becoming fathers in what they perceive as less than ideal circumstances of dealing with negative social perceptions against sex work. This experience is echoed in other studies that have focused on parenting among sex workers and their partners [1, 21, 23]. Our findings further reveal how fatherhood and masculinity is framed within socially constructed meanings of what it means to be a father, with the provider role privileged over other fatherhood roles [33]. The social context in which men become fathers shaped how men approached their different roles and dealt with challenges [12].

We found that men viewed fatherhood as a public role which was associated with respect and pride within the society, while engaging in paid sex is a secretive affair better hidden from the public. The two roles seemed incompatible, and posed a significant challenge to men’s construction of identity. The stigma that is reported by some men resulted from the identity of the work of their partners; a feature found in other literature on sex workers [15, 22]. The hesitancy of men to accept parental responsibility for children born to sex workers is a reflection of the prejudice against sex workers that is so prevalent in many societies including Uganda [34]. The marginalization and exploitation of FSW is manifested in the way men exhibit their private sexual desires but then feel shamed when pregnancies result [22]. Thus our findings highlight that FSW bring significant benefits to the relationships with male partners which helps the couples to build relationships based on the normal societal expectations. Roles that enhance a respectable status in society such as fatherhood (and motherhood) are recognized but those that might bring disrepute to the relationship (for example sex work) are look down upon. We noted that men appreciated and acknowledged the benefits they derived from the sex workers and were, at least for a time, able to commit to the relationship, but they—as the wider society—struggled with the negative perceptions of sex work [22, 34, 35]. This double standard where men enjoy sexual services and yet are reluctant to embrace the responsibility as father when FSW get pregnant undermines co-parenting among this population, and contributes to child neglect and vulnerability [1, 20, 24]. The marginalization of sex workers at a structural level, manifested through strict laws that criminalized sex work in Uganda, result in women not only being susceptible to exploitation [26], but having unwanted pregnancies due to limited access to contraceptives [3]. This further complicates the performance of parenting roles these mothers and fathers.

However, despite some prejudices men hold against FSW which result in men abandoning parental responsibility, the men in our study who played some part in the lives of their children demonstrate that there is potential to target men who father children with FSW and improve their participation in parenting [36, 37]. This provides a platform for children to benefit from their father’s involvement in their lives [38]. Female sex workers that offered financial support to their male partners did so partly to gain acceptance and offer their children the access to their father’s lineage, which is important in patrilineal societies [15]. FSW were also keen to embrace the identity of mothers in a stable family environment. Providing support to the father of their child facilitated this process [22].

Our findings also show that fathering a child with a FSW can be a complex affair as evidenced in the case of Musa whose status as a father of twins was abruptly ended or be loaded with doubts as in the case of Donald. This gives men an excuse to avoid responsibility over children born to partners that are sex workers [21]. However, in contrast, the reaction of men like Patrick and Musa who expressed deep disappointment at losing their paternity contradicted some of the previous literature that seems to suggest that men will find the easiest excuse to disassociate themselves from children if the circumstances are challenging [39].

The discomfort among financially disadvantaged men who could not provide for their children and families has been noted before among urban based men in East Africa [40]. However, having more money than their partners did not translate into women taking over leadership roles within their families. The men retained this position with their FSW partners being tolerant and patient with them to reclaim their roles as providers [41]. This ability to retain their powers even though they may have had challenges in providing allowed some of the men to continue with practices that were normally associated with building peer reputation. This seemingly deliberate move to keep men in power position fits within the social construction of gender power that places men in particularly privileged positions [42, 43]. It also demonstrates the power that masculinities gives to men that even when women are the providers, women remain uncomfortable identifying with such a masculine role [44].

In recent years, there have been calls for the fatherhood role to be defined more broadly and for credit to be given to fathers who are involved in the lives of their children, beyond being a provider, even if they may not meet the standard expected of fathers in different settings [45–47]. Our findings show how men in disadvantaged social positions go beyond the provider role and build a supportive environment between themselves and their partners thus stabilizing the family [48]. Interventions that encourage involvement of fathers have documented success when fathers are positively engaged [49].

In most of the cases described in this paper, the women had welcomed the involvement of the fathers of their children partly because most women had previously experienced being abandoned by men upon learning of pregnancy [15]. When men stood with their partners despite the negativity surrounding sex work, women reciprocated in ways that benefitted the men. Those men that needed HIV treatment for example were able to access treatment and support from their female partners which helped the men to participate in the lives of their children without worrying about their health. This is an important finding that builds on previous work we have conducted with a similar population [18], and from other studies in the East African region [19, 50].

The study has some limitations that ought to be taken into consideration to help interpretation. First in selecting the sample, we depended on the good will of the female sex workers who were our gate way to reaching the men. It is possible that the men we eventually recruited could have been those in a healthy relationship with their partners thus missing out on a wider variety. We are however, aware of the difficulty in recruiting men in relationships with sex workers [51]. Secondly, the partners of the men in this sample were all part of a research and intervention programme whose membership to such programmes could have influenced their lived experiences. It is possible that fathering practices of other men might have been different.

## Conclusion

Men who have children with FSW face hurdles fitting within the social construction of ideal fatherhood and masculinity. We found that the initial reactions of the men in this study to doubt pregnancies even when they admit to condomless sex with FSW is a typical reflection of the stigma associated with sex work. However, once they overcame their prejudices against sex work due to the sexual pleasure and satisfaction, money and financial support that accrued from relating with a FSW, as well as the acceptance from family, accepting responsibility conferred a high social status and positive identity to the men, resulting in them participating in fatherhood roles. Nonetheless, the predominance of the provider role as standard measure of fatherhood both at family and societal levels meant that men felt inadequate in their role, since many could not fulfil it due to financial constraints. There is need for parenting and economic strengthening interventions tailored to the circumstances of men who father children with female sex workers and their partners.
